# Treatment-resistant psychotic symptoms and the 15q11.2 BP1–BP2 (Burnside-Butler) deletion syndrome: case report and review of the literature

**DOI:** 10.1038/s41398-020-0725-x

**Published:** 2020-01-28

**Authors:** Martilias Farrell, Maya Lichtenstein, Matthew K. Harner, James J. Crowley, Dawn M. Filmyer, Gabriel Lázaro-Muñoz, Tyler E. Dietterich, Lisa M. Bruno, Rita A. Shaughnessy, Tamara F. Biondi, Stephan Burkholder, Jane Donmoyer, Jonathan S. Berg, Jin Szatkiewicz, Patrick F. Sullivan, Richard C. Josiassen

**Affiliations:** 1 Department of Genetics, University of North Carolina at Chapel Hill, Chapel Hill, NC USA; 2 Department of Neurology, Geisinger Health System, Wilkes Barre, PA USA; 3 Translational Neuroscience, Conshohocken, PA USA; 4 Center for Medical Ethics and Health Policy, Baylor College of Medicine, Houston, TX USA; 5 Wernersville State Hospital, Wernersville, PA USA; 6 Department of Psychiatry, University of North Carolina at Chapel Hill, Chapel Hill, NC USA; 7 Department of Medical Epidemiology and Biostatistics, Karolinska Institutet, Stockholm, Sweden

**Keywords:** Diagnostic markers, Diagnostic markers, Personalized medicine, Personalized medicine, Personalized medicine

## Abstract

The 15q11.2 BP1-BP2 (Burnside-Butler) deletion is a rare copy number variant impacting four genes (*NIPA1, NIPA2, CYFIP1, and TUBGCP5*), and carries increased risks for developmental delay, intellectual disability, and neuropsychiatric disorders (attention-deficit/hyperactivity disorder, autism, and psychosis). In this case report (supported by extensive developmental information and medication history), we present the complex clinical portrait of a 44-year-old woman with 15q11.2 BP1-BP2 deletion syndrome and chronic, treatment-resistant psychotic symptoms who has resided nearly her entire adult life in a long-term state psychiatric institution. Diagnostic and treatment implications are discussed.

## Introduction

Neurodevelopment is a complex set of biological processes that result in the orderly development and maturation of the nervous system^1^. Any disruption in this tightly orchestrated chain of events may lead to altered brain development and to an abnormal neurodevelopmental phenotype^2–4^. Factors that can disrupt neurodevelopment are not fully delineated, but a significant proportion of neurodevelopmental risk is attributed to copy number variants (CNVs)^5,6^. CNVs are structural mutations that occur when genomic regions are duplicated or deleted compared to the reference genome^7^. Several large, rare CNVs have been robustly associated with increased risk for neurodevelopmental disorders (NDDs) including intellectual disability (ID), developmental delay (DD), autism spectrum disorder (ASD), attention-deficit/hyperactivity disorder (ADHD), and multiple congenital anomalies^8,9^. Of all childhood cases referred for chromosomal microarray analysis (CMA), ~10% carry a pathogenic CNV^10,11^. A recent paper^12^ summarized CMA results from >10,000 patients presenting with ID, DD, multiple congenital anomalies, and/or ASD; the 15q11.2 BP1–BP2 deletion was among the most common pathogenic CNVs found. Interpretation of these CNVs is complicated by variable phenotypic expression, incomplete penetrance, and limited data^13^. CMA investigations of NDDs have yielded important data regarding etiological significance and their importance for diagnostic and clinical management.

Although the utility of CMA is established for childhood NDDs, its application in adult psychiatric practice has been slow^14,15^ despite data suggesting the neurodevelopmental origins of schizophrenia^16,17^. Currently, ~10 rare CNVs have been associated with schizophrenia (including the 15q11.2 BP1–BP2 deletion). As we argue^23^, we need more data on the clinical characteristics and longitudinal treatment outcomes of schizophrenia patients with these rare CNVs^18–21^. Importantly, most CNVs associated with schizophrenia also confer risk for DD, ID, ASD, and/or ADHD, but the implications for nosology, diagnosis, and clinical management require more study^18–21^.

More data is needed to support clinical decision-making for adults with co-occurring pathogenic rare CNVs and severe psychiatric disorders^18,22^. Large studies of these CNVs are optimal (there are consortia for 22q11, 16p11, etc), but accruing sizeable samples requires a global effort, considerable expense, and years of work^22,23^. To improve our knowledge base more rapidly, Sullivan and Owen^23^ proposed clinical crowd-sourcing, “a systematic effort be launched to obtain high quality case-reports and case series that would begin to inform reasonable therapeutic clinical management of these complex schizophrenia cases.” Several recent examples in the literature^24–26^ underscore the potential value of making more widespread use of CMA in such instances.

Accordingly, we present here a detailed developmental/psychiatric history, description of longitudinal course, and summary of therapeutic efforts for a woman who, from childhood onward, presented with psychotic symptoms nonresponsive to multiple antipsychotic medications (including clozapine). CMA results revealed a pathogenic 15q11.2 BP1-BP2 deletion (Burnside-Butler) syndrome^27,28^. Our goal in presenting this case summary is to encourage clinicians to consider the possibility that atypical clinical presentations in a context of chronically severe and largely refractory clinical responses might have an identifiable genetic origin (with potential treatment implications). In such cases, it would be worthwhile to obtain genetic testing for rare CNVs, some of which may be medically valuable for the patient, their family, and their clinicians.

## Case report

Ms. A is a 44-year-old woman of European ancestry who participated in our genomic study focusing on patients with chronic psychotic symptoms non-responsive to ≥ 3 trials of antipsychotic medication of adequate dose and duration (i.e., treatment-resistant psychotic symptoms, TRS)^29^. Those who also fail clozapine treatment are described as having ultra-TRS^30^. Ms. A was selected for study due to multiple atypical features, including childhood-onset psychotic symptoms and ultra*-*TRS. All study procedures were approved by the Committee for the Protection of Human Subjects at Drexel University College of Medicine and Ms. A provided written informed consent. The protocol allowed for return of results and subject re-contact. All aspects of her participation in this study were discussed with her treating psychiatrists and patient advocate.

### Developmental and psychiatric history

Table 1 provides a detailed overview of significant life events, development, and course of illness (based on review of extensive medical records and interviews with her treatment team and biological mother). The mother was primiparous and in her late teens at the time of delivery. She denied consuming ethanol, nicotine, or illicit drugs during pregnancy. Ms. A was born at term without complications.

**Table 1:** Life course chart for Ms. A

Age	Life event	Clinical notes
0	Birth	Without complications, mother was anemic.
1–5 years	Early childhood	Developmental delays in walking and talking, severe temper tantrums and possible auditory hallucinations.
6–7 years	Mother develops significant medical illness, parents’ divorce. Placed in foster care. Began school.	Developmental delays; learning disability; auditory hallucinations, agitation, suicidal behavior; special education classes; IQ 70–75; severe behavioral problems.
7–9 years	Public school and day treatment	Enrolled in special education classes. Placed in different foster care homes with occasional stays with biological mother. Haloperidol started. Transfer to residential treatment program. All psychiatric medications discontinued, diagnosis of schizotypal personality disorder and pervasive developmental delay.
9–12 years	Admitted to long-term school-hospital for children with mental health problems.	Worsening behavioral problems; suicidal thoughts; auditory hallucinations. IQ of 65. Diagnosed with schizophrenia (chronic undifferentiated type), borderline intelligence, and atypical seizures. Haloperidol restarted.
12–14 years	Discharged to mother	Severe psychiatric symptoms persist.
14–17 years	Foster care placement and admission into intensive special education day program.	Auditory hallucinations; sexual preoccupation; perseveration; poor impulse control; anxiety; immature behaviors; suicidal ideation and behavior. Required 1:1 observation. Possible seizure disorder with abnormal spiking in EEG noted. Diagnoses of schizophrenia and possible absence seizures.
17–18 years	“Aged-out” of treatment program, transferred to community hospital	Transferred to a community psychiatric hospital. Diagnosis: Schizophrenia, residual, chronic; possible absence seizures.
18–25 years	Admitted to long-term hospital	Severe behavioral and psychotic symptoms; prominent agitation and aggression; often on 1:1 suicide prevention. GAF scores in 30–40 range.
26–34 years	Community-based care	Numerous (18) community short-term hospitalizations.
35–44 years	Admitted to long-term hospital	Continuing pattern of severe behavioral and psychotic symptoms; severe agitation and aggression. On nearly continuous 1:1 for suicide prevention for 2 years. Diagnoses include schizoaffective disorder (bipolar type), borderline intellectual functioning; possible seizure disorder. GAF scores in 30–40 range.
41 years	2 seizure-like episodes	Unenhanced CT scan of brain unremarkable.

Ms. A evidenced DD (e.g., walking, talking milestones), had recurrent severe temper tantrums, extensive conversations with “imaginary friends”, and possible auditory hallucinations dating to age 5. Developmental and behavioral disturbances became increasingly apparent when Ms. A started elementary school, which resulted in her being placed in foster care and special education classes. From age 9–12, she resided in a child/adolescent state-sponsored institution. In this institution she was described as easily frustrated, leading to temper tantrums and often “regressing to the level of a 3-year-old”. The medical record describes “bizarre giggling spells or making sounds like animals”, and visions of angels who “said nice things to me”. She often expressed thoughts of killing herself, engaged in high-risk self-injurious behavior, and was commonly found under her bed pretending she was dead.

When the child/adolescent state-sponsored institution was closed, she was transferred to foster care and was in special education classes to age 18 when she aged out of these services. She was admitted to an adult, long-term state psychiatric institution where she resided continuously from age 18–26. Due to a state mandate for institutional downsizing, she was discharged to a community residential facility where she resided from ages 26–34. During this time, she required 18 psychiatric hospitalizations and was eventually readmitted to a state hospital where she has resided continuously from age 34–44.

### Medication history

**Fig. 1:**
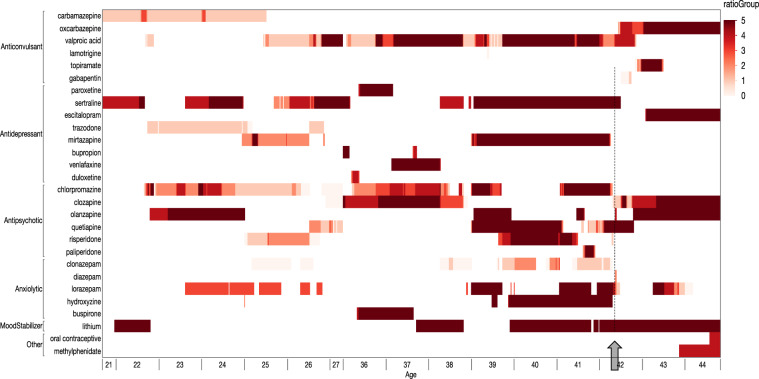
Summary of psychotropic medication given to Ms. A from ages 21–27 and 33–44. Using a hospital-based electronic pharmacy record, the dosage of each psychiatric medicine per week was tabulated. The *X*-axis is age with each year comprising up to 52 thin, weekly slices. The *Y*-axis shows broad drug classes and the vertical sections within each class show the specific medications. The color of each vertical slice depicts the ratio of the prescribed amount of drug to “defined daily dose” specified by the World Health Organization for each drug (from very light to very dark red with the two darkest colors showing a ratio >1 or exceeding that defined daily dose). Ms. A has received substantial trials of: clozapine, three other atypical antipsychotics, and multiple typical antipsychotics (per history, only chlorpromazine shown); lithium; four anticonvulsants; five antidepressants; and multiple anxiolytics. Arrow and dotted line indicates the approximate time of re-conceptualization.

Ms. A first received haloperidol (20 mg daily) at age 7. Prior to age 18, she had been treated with haloperidol, fluphenazine, thioridazine, thiothixene, trifluoperazine, chlorpromazine, mesoridazine, and loxapine. Figure 1 summarizes the duration and dosages of psychotropic medications prescribed during extended inpatient hospitalizations as an adult: clozapine, three other atypical antipsychotics, multiple typical antipsychotics; lithium; four anticonvulsants; five antidepressants; and multiple anxiolytics. Clozapine was initiated at age 35 with stable WBC and ANC measurements for approximately 3 years, but with little to no clinical benefit. At age 38, clozapine was discontinued due to an abrupt drop in absolute neutrophil count (4.0 to 2.3/mm^3^), but successfully restarted at age 41 with minimal efficacy. Polypharmacy of increasing dosages provided little clear clinical benefit.

### Past medical/surgical history

Type 2 diabetes mellitus, hypertension, chronic obstructive pulmonary disease, dependent edema, gastroesophageal reflux disease, hypothyroidism (in remission), chronic constipation, past history of pleural effusion of right lung (resolved), acute renal failure attributed to vancomycin and lithium toxicity; surgery to correct strabismus.

### Family history

Maternal grandmother was diagnosed with schizophrenia and committed suicide.

### Laboratory investigations

Computed tomography of the head without contrast at age 41 was unremarkable. An abnormal EEG was mentioned in her progress notes but no report was available. As shown in Table 1, Ms. A’s cognitive functioning was assessed^31^ between ages 6–14, and documented ID with full scale IQ scores from 65–75. We assessed Ms. A’s cognitive functioning at age 44 with the Wechsler Adult Intelligence Scale III^32^, and found verbal IQ of 62, performance IQ of 56, and full scale IQ of 61 (Table S1). These values are consistent with ID and appear approximately stable over time.

### Physical examination

Ms. A was examined by a behavioral neurologist (ML). She presented as an overweight woman with straight light hair, wide mouth, crooked teeth and maxillary prognathism, and was casually dressed in loose clothing. She was cooperative with the examiner, and child-like in behavior and affect. She perseverated about food, her appetite, and stomach sounds, and became anxious several times that she might miss a meal. She spoke clearly without dysarthria and made good eye contact. She described her mood as “pretty good”. She had an occasional stare; it was not clear whether it was behavioral, an absence seizure, or if she was responding to internal stimuli. She denied auditory or visual hallucinations.

She scored a 25/30 on the Mini Mental Status Examination^33^. Her attempt to draw a clock face showed deficient planning and misplaced hands. She was able to identity 2/5 emotions correctly on the Penn Facial Emotion Recognition task^34^. Her language was fluent with no surface dyslexia; however, she displayed concreteness and lack of attention to detail when describing a common image (a beach picture).

Neurological examination was unremarkable except for mildly increased tone with augmentation in the wrists, brisk symmetric deep tendon reflexes with mute planter response, and mild action tremor without dysmetria on finger-to-nose testing. She had a slightly wide-based gait, was unable to tandem walk, and had difficulty standing with her feet close together.

### Genetic analyses

Genomic DNA was extracted from a peripheral venous blood sample and genome-wide SNP genotypes obtained using Illumina Global Screening Array (v1.0, GSA-24z1-0_C1) per standard protocols. CNVs were called using PennCNV^35^. This research-grade analysis identified a large, high-confidence one-copy deletion on 15q11.2, a region robustly associated with risk for multiple neurodevelopmental disorders. The presence of a clinically significant, rare pathogenic CNV was confirmed using a clinical-grade Agilent comparative genome hybridization array in a CLIA-certified lab (Allele Diagnostics, Spokane WA): a deletion CNV on chr15:22.82–23.09 Mb (hg19 genome build), also known as 15q11.2 BP1-BP2 **(**Burnside-Butler) deletion syndrome^27,28^. The clinical features found in Ms. A that were consistent with those reported in Burnside-Butler syndrome included delayed psychomotor and speech development, ID, abnormal impulsive behavior (including pica), psychotic symptoms, and possible seizures. Other features included a history of recurrent upper airway infections, strabismus, and irregular dentition. CLIA testing found an additional variant of uncertain significance (698 kb deletion at 2q12.3 from chr2:108.54–109.24 Mb, hg19) containing six protein-coding genes (*SULT1C4*, *GCC2*, and *LIMS1* are expressed in brain and *SLC5A7*, *SULT1C3*, and *SULT1C2* are not). None of these genes have been associated with any psychiatric disorder in the most recent rare CNV or genome-wide association studies.

### Clinical course

Just prior to Ms. A’s participation in the genetic study, her treatment team began a reconceptualization of her diagnosis and treatment. Pharmacologically, the goal of therapy was to remove unnecessary medications that may have contributed to behavioral dysregulation or akathisia (i.e., minimize benzodiazepines and typical antipsychotics). She appeared to benefit from these interventions; however, she could only partly engage in behavioral interventions due to inattention, obsessive thoughts, and limited cognitive processing. She was given a trial of a stimulant which was well-tolerated and led to improved attention. Because of repeated premenstrual mood symptoms, she was started on an oral contraceptive which she and staff believed reduced irritability and anxiety.

Behavioral interventions centered on the goals of reinforcing adaptive skills, addressing frustration with gestalt therapeutic techniques, and providing a consistent environment (seemingly minor disruptions to her daily routine often led to suicidality and clinical worsening). Ms. A engaged daily with her provider to establish therapeutic alliance and to reinforce basic coping strategies. Given her considerable anxiety about change and reliance on 1:1 observation, it was felt that perhaps the best strategy was to explain to the patient that 1:1 would be a long-term intervention and unlikely to change for a considerable amount of time. Therapy focused on strategies to reinforce independence such as seeking out support, verbalizing her struggles, and reflecting on positives and negatives of her daily activities. Gradual tapering of 1:1 was initiated (e.g., attending a group or activity without the 1:1 staff), followed by increasing the distance from 1:1 staff, and being seated in the dayroom without staff for incremental periods of time. Although there continue to be episodes of emotional outburst (e.g., yelling and head-banging leading to 1:1 observation and emergency medication), these were greatly attenuated. At the time of this writing, Ms. A was regularly attending group recreational outings and hospital discharge was being planned.

With Ms. A’s permission, the treatment team was informed of the 15q11.2 (BP1-BP2) deletion. The treatment team stated that knowledge of the pathogenic rare CNV provided useful early support for their re-conceptualization of Ms. A’s primary diagnosis as more pervasive DD and ID than primarily psychosis.

## Discussion

As part of our ongoing investigation into the genomics of treatment-resistant psychotic symptoms^36^, we performed a comprehensive genetic analysis of Ms. A. She had been diagnosed with a psychotic disorder since childhood and over the decades this diagnosis had become the dominant focus of her clinical management, even though multiple, severe neurodevelopmental symptoms contributed to her highly complex clinical presentation. The discovery of a pathogenic rare CNV and a thorough evaluation of her symptoms raised the possibility that many of the features present in Ms. A. were consistent with those reported in the 15q11.2 BP1-BP2 deletion (Burnside-Butler) syndrome.

**Fig. 2:**
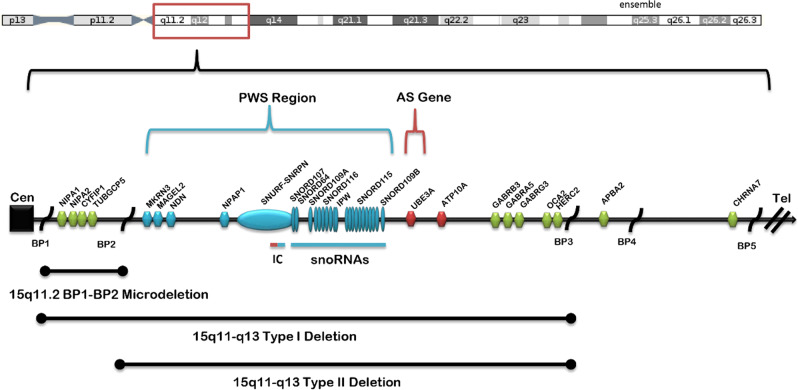
Ideogram of the 15q11–q13 region taken from Butler^44^. Chromosome 15q breakpoints (BP1, BP2, BP3, BP4, BP5) are shown along with locations of three 15q deletions including 15q11.2 BP1-BP2. The patient described in this case report has a deletion between BP1-BP2 that affected copy number of *TUBGCP5*, *CYFIP1*, *NIPA2*, and *NIPA1*. “Type I” and “Type II” deletions at bottom of figure refer to PWS/AS. Abbreviations: PWS = Prader-Willi Syndrome, AS = Angelman Syndrome, BP1-5 = breakpoints 1–5, Cen = centromere, Tel = telomere. (Used with author permission).

As shown in Fig. 2, the proximal long arm of chr15 has five breakpoints (BP1-BP5), which mediate increased rates of CNVs within this region. The 15q11.2 BP1-BP2 deletion lies in a ~500 kb region between breakpoints BP1 and BP2. The 15q11.2 BP1-BP2 deletion contains four highly conserved protein-coding genes *(NIPA1, NIPA2, CYFIP1*, and *TUBGCP5)*. Each of these genes has a reported behavioral finding associated with pathogenic variation^27,28^. *NIPA1* is associated with autosomal dominant hereditary spastic paraplegia and postural disturbance when mutated. It is a magnesium transporter and is highly expressed in the brain. *NIPA2* is a renal magnesium transporter and, when mutated, causes childhood absence epilepsy. The lack of severity of the Burnside-Butler deletion compared to these gene specific phenotypes is likely due to the presence of a functional copy. *TUBGCP5* is also highly expressed in brain and has been associated with ADHD and obsessive-compulsive disorder (OCD). Finally, *CYFIP1* plays an important role in neuronal cytoskeletal remodeling, and reduced expression of *CYFIP1* has been implicated in the dysregulation of schizophrenia- and epilepsy-associated gene networks^37^. The CYFIP1 protein interacts with the fragile X mental retardation protein (the absence of which causes fragile X syndrome). A very recent imaging study of 15q11.2 BP1-BP2 deletion carriers^38^ found abnormal white matter microstructure similar to that previously reported in fragile X syndrome, suggesting a role of *CYFIP1* in the 15q11.2 deletion phenotype. A study^39^ of neurons derived from patients with the 15q11.2 BP1–BP2 deletion showed abnormalities of dendritic spine formation.

Estimates of the prevalence of the 15q11.2 BP1–BP2 deletion in schizophrenia cases range 0.14–0.65%^19–21,40^. Individuals with 15q11.2 BP1-BP2 deletion can present with a wide range of clinical findings. ID and language delays are found in greater than two-thirds of cases, along with autism, behavioral problems, poor coordination, ataxia, and/or congenital anomalies. Psychiatric findings can include schizophrenia, oppositional defiant disorder, OCD, and dyslexia^38,41^. However, within the clinical setting most diagnosed cases have not been systematically evaluated with comprehensive clinical, medical, and behavioral assessments. A literature review and summary of clinical findings from 200 individuals with the 15q11.2 BP1–BP2 deletion identified: DDs (73%) and language impairment (67%); dysmorphic ears (46%) and palatal anomalies (46%); writing (60%) and reading (57%) difficulties, memory problems (60%), and verbal IQ scores ≤ 75 (50%); and abnormal brain imaging (43%)^42^. Other less frequent features were seizures/epilepsy (26%), ASD (27%), ADHD (35%), and schizophrenia/paranoid psychosis (20%). Interpretation of this syndrome in a clinically useful way is particularly challenging due to highly variable phenotypic expressivity, incomplete penetrance, and limited data. Not all individuals carrying the deletion will present with overt symptoms. A recent study^43^ found mild cognitive impairments in individuals with the deletion who did not meet criteria for any psychiatric diagnosis. The presence of a specific rare CNV does not necessarily imply that it played a causal role and rare CNV carriers can be only mildly affected or unaffected. Nevertheless, establishment of the Burnside-Butler syndrome as a risk factor in schizophrenia is an important development.

There are no comprehensive guidelines for treating the psychiatric component of 15q11.2 BP1-BP2 deletion syndrome^27^. Butler^44^ recently proposed dietary magnesium supplementation as two of the four genes impacted by 15q11.2 BP1-BP2 CNV deletions (*NIPA1* and *NIPA2*) are magnesium transporters. Magnesium is required by hundreds of proteins critical for cellular functions like energy expenditure, protein synthesis, DNA transcription, and muscle and nerve function. Butler proposed that magnesium supplements could be a treatment option and provided limited anecdotal information as support. Based on this suggestion, Ms. A was prescribed magnesium (450 mg daily) by her treatment team (her baseline serum magnesium was 1.6 mEq/L, normal range 1.5–2.5 mEq/L). Unfortunately, the efficacy of this supplementation is currently unknown in this case.

Clinically, identification of the 15q11.2 BP1–BP2 (Burnside-Butler) deletion had unexpected beneficial impact. First, while the treatment team had already concluded that a different course of treatment was warranted, knowledge of the rare CNV led to a more informed diagnostic understanding of this complex case, and supported their reconceptualization of a treatment focus more on DD-ID-ADHD than psychosis. The presence of this rare CNV is now part of her medical record. The new genetic information stimulated conversation among treatment team members about ordering “client-specific genetic testing” for complex cases. Various diagnostic and treatment implications were discussed which raised conjecture about new therapeutic avenues that might replace therapeutic nihilism. Second, the presence of 15q11.2 BP1–BP2 deletion stimulated interest in exploring a more appropriate therapeutic setting. Long-term services for ID/DD are substantially different from psychiatric services, and the treatment team launched an exploration of programs that might be more beneficial. Third, return of genomic findings to the biological mother offered her the opportunity to re-frame her understanding of the factors contributing to her daughter’s illness, and hopefully reduced her burden of stigma and guilt.

Investigation of rare CNVs is beginning to provide a novel biologically defined entry point for studying the underlying genomic architecture of various NDDs as well as individual psychiatric cases of diagnostic and/or management complexity. The etiologic implications of rare CNVs are intriguing and individual case findings raise possibilities for enriched diagnostic understanding and new treatment avenues. However, the interpretation of these findings is challenging even for specific pathogenic CNVs. CNVs confer probabilistic and not deterministic risk, and considerable caution is warranted as not to overestimate etiologic, diagnostic, and clinical importance. In the case of individuals with TRS or ultra-TRS under psychiatric care, it seems possible that routine CMA testing could become an established practice in the near future. There is a strong case to be made for a genetic workup in select individuals, particularly those with severe psychotic disorders and therapeutic non-response.

## Supplementary information

Supplementary Material
