# Supplementary material for: Treatment-resistant psychotic symptoms and the 15q11.2 BP1–BP2 (Burnside-Butler) deletion syndrome: case report and review of the literature
Source: Transl Psychiatry. 2020 Jan 28;10:42. doi: 10.1038/s41398-020-0725-x (PMC7026068; doi:10.1038/s41398-020-0725-x)
Supplement: Supplementary file 1 — Supplementary Material [file 41398_2020_725_MOESM1_ESM.doc]

**Table S1:** Cognitive assessment of Ms A at age 44.

| **Subtest** | **Scaled Score** | **Subtest** | **Scaled Score** |
| --- | --- | --- | --- |
| Vocabulary | 4 | Picture Completion | 4 |
| Similarities | 4 | Digit Symbol | 3 |
| Digit Span | 3 | Picture Arrangement | 2 |
| Information | 4 | Block Design | 3 |
| Comprehension | 3 | Matrix Reasoning | 5 |
| Letter Number | NA | Object Assembly | NA |
| Arithmetic | 1 | Symbol Search | NA |

Wechsler Adult Intelligence Scale III. Verbal IQ = 62, Performance IQ = 56, Full Scale IQ = 61.
